# The Influence of Phase Change Materials on the Properties of Self-Compacting Concrete

**DOI:** 10.3390/ma6083530

**Published:** 2013-08-15

**Authors:** María Fenollera, José Luis Míguez, Itziar Goicoechea, Jaime Lorenzo, Miguel Ángel Álvarez

**Affiliations:** 1 Department of Design in Engineering, Industrial Engineering School, University of Vigo, Vigo 36310, Spain; E-Mail: igoicoechea@uvigo.es; 2 Department of Mechanical Engineering, Machines and Thermal Engines and Fluids, Industrial Engineering School, University of Vigo, Vigo 36310, Spain; E-Mails: jmiguez@uvigo.es (J.L.M.); alvarezfeijoo@cud.uvigo.es (M.Á.Á.); 3 Department of Materials Engineering, Applied Mechanics and Construction, Industrial Engineering School, University of Vigo, Vigo 36310, Spain; E-Mail: jaimelorenzo@uvigo.es

**Keywords:** self-compacting concrete, phase change materials, dosage, mechanical properties, structural concrete panels, mix design

## Abstract

The aim of this paper is to research new thermally-efficient concrete walls, analyzing the mechanical behavior of a self-compacting concrete to manufacture an uncoated solid structural panel, with the incorporation of a micro-encapsulated phase change material as additive. Different dosages are tested and mechanical properties of the product obtained from the molding of concrete specimens are evaluated, testing mechanical compressive strength, slump flow, and density. The results reveal the optimum percentage of additive in the mixture that enables compliance with the technical specifications required by the product to be manufactured. A test is also performed for measuring the thermal conductivity for the optimal sample obtained and it evidences the reduction thereof.

## 1. Introduction

For existing industries, the term sustainability is paramount when dealing with innovation. The design and development of sustainable products is a factor of increasing importance in industrial engineering. The construction industry, the main consumer of material resources and energy, has great potential in the development of new efficient materials to reduce energy consumption in buildings, so materials research aimed at sustainable construction and its applications appear as the engine of economic competitiveness.

The research project of which the results are presented in this paper optimizes the design and construction of buildings by improving thermal insulation or storage. In order to achieve this, it is based on the following combination: one the hand, on the fact that the phase change materials (PCMs) are latent heat thermal storage materials (where energy transfer occurs when the material changes phase at almost constant temperature), and on the other hand, on heat capacity and high density of concrete. This optimization implies integrating microencapsulated PCM into self-compacting concrete (SCC), thus increasing the capacity of thermal storage of the building envelope. 

SCC has been selected due to its several advantages: high performance of both fresh and hardened concrete (high flowability and segregation resistance, low porosity, high strength and durability, *etc.*); wider applications (components and structures with complicated shape and highly congested by steel reinforcements); money saving (increased works’ speed and reduced costs for energy, equipment and workmanship); enhancement towards modernization of construction process; environment protection due to high consumption of industrial by-products and improved working environment by reduced noise and health hazards [1,2].

The thermal energy storage in buildings can be achieved mainly by sensible heat or by latent heat (for example, including PCMs). The main advantage of the latent heat is the high storage density at small temperature intervals. PCMs have been classified into three major groups: organic, inorganic, and eutectic [3]. Organic PCMs have been chosen from the beginning as the preferred means of research because of their important advantages and their manageable disadvantages [4,5]. 

To integrate PCMs in building, various techniques have been investigated, like immersion processes or macrocapsules; however, because of their limitations, these techniques have not been well received in the market [6]. Recent advances in microencapsulation technology make it suitable for introducing organic PCMs (preferably paraffin wax) in thermal energy storage systems [7]. Microcapsules are small PCM particles forming the core, coated with another material that forms a thin protective insulating film, with the great advantage that it is easier to manipulate the PCM, in addition to tolerating volume changes, reducing the reactivity of the paraffin wax with the outside and increasing the heat transfer area [8]. 

The use of PCMs in building materials for thermal storage has been extensively studied since the 1980s [9,10,11] and the number of articles relating to the integration of PCMs in walls of buildings has increased enormously in the last five years [12]. Tyagi *et al.* [13] made a complete review of the major research in this area. The most relevant to this investigation are contributed by authors such as Cabeza *et al.* [14] carrying out tests in conventional concrete cubicles, one with 5% (by mass of concrete) of PCM microencapsulated in powder (melting point 26 °C and phase change enthalpy 110 kJ/kg) and another without PCM, analyzing the effects on the internal temperature of the cubicles and thermal inertia, and found that thermal inertia is improved and indoor temperatures are reduced in the cubicle with PCM, thus serving structural purposes (compressive strength 25 MPa after 28 days). 

Hunger *et al*. [15] test a set of SCC mixes, directly adding different percentages (1%, 3%, and 5% by mass of concrete) of PCM microencapsulated in powder (melting point 23 °C and enthalpy of phase change 100 kJ/kg), whereby the thermal conductivity of the concrete is reduced as the percentage of PCM increases, as well as significant loss of compressive strength and 3% of PCM, mainly due to the breakage of the microcapsules. 

The same microencapsulated PCM was used by Entrop *et al.* [16] in concrete floors in order to help heat homes during the night by using solar radiation as a source of thermal energy stored in the floors during the day, the mix composition is used by Hunger *et al*. [15] with 5% PCM by mass of concrete, except PCM incorporate in the last stage of the mixing process to prevent rupture of the microcapsules, thus minimizing mechanical stress. 

Finally, Meshgin *et al.* [17] verified that adding 15% (by mass of concrete) of PCM microencapsulated in wet cake (melting point 28 °C and enthalpy of phase change 180 kJ/kg) to the mortar, reduces the mechanical properties of the concrete but its heat capacity improves. These results are compared with the other samples containing different percentages and rubber particle size.

The main objective of this paper is to contribute to existing knowledge in the use of PCMs in SCC, in particular, the new structural precast concrete panel must have the following characteristics: density of about 2400 kg/m^3^ and thermal conductivity of 1.5 W/m °C. The concrete for the manufacture of the panel is a HA-40/AC/12/IIa, a SCC with a characteristic strength of 40 N/mm^2^, fluid consistency, 12 mm maximum aggregate size and IIa environment. The chosen PCM is an aqueous dispersion of a mixture of formaldehyde-free microencapsulated paraffin. 

The determination of the maximum percentage of PCM to be added to mass concrete is mainly limited by the mechanical strength of the structural panel to be obtained. Therefore, a study of mixes with the following PCM percentages by mass of cement is performed: 0%, 5%, 10%, 15%, 20%, and 25%, higher percentages are discarded because of potential problems with mixture. The research cited above shows loss of mechanical strength in the concrete and breakage of the encapsulation of the PCM, followed by exudation and loss thereof, and segregation of the PCM to the upper surface of the SCC and poor dispersion in the mass. 

To evaluate these aspects as well as the mechanical properties of fresh SCC, the following actions have been carried out:

- Compressive strength: the test results are primarily used to determine that the concrete mixture as delivered meets the requirements of the specified strength in the job specification.
- Slump flow test: evaluates the fluidity and flow rate of SCC in the absence of obstructions.
- Segregation of the PCM to the top of the concrete and/or poor dispersion of PCM: to observe the fracture surfaces of the test specimens that were tested to determine the mechanical strength, in case the dispersion was so poor, or segregation so great that you would see them with the naked eye.
- Encapsulation breakage: add PCM in aqueous dispersion and incorporate it into the final stage of the mixing process.
- Measurement of densities.

## 2. Experimental Method

### 2.1. Materials 

Materials available in the locality of O Porriño of Pontevedra (Spain) have been used in order to design concrete HA-40/AC/12/IIa, such materials are similar to those that the company producing panels normally uses, and new materials have been added based on availability of their regular suppliers and their characteristics listed below:

- Cement: CEM I-52.5 R is the cement type used, a standard component in high quality concrete, according to UNE-EN-197-1: 2011 [18]. It has a high initial mechanical strength, which facilitates handling and demolded at early stages.
- Filler: the filler used is a calcareous Filler A. Its primary mission is to increase the viscosity of the mixture, to stabilize the water within the concrete, and to increase the surface tension and the cohesion thereof. It was selected for its fineness (maximum size of 60 µm) according to UNE-EN 12620: 2003 + A1: 2009 [19].

**Figure 1:**
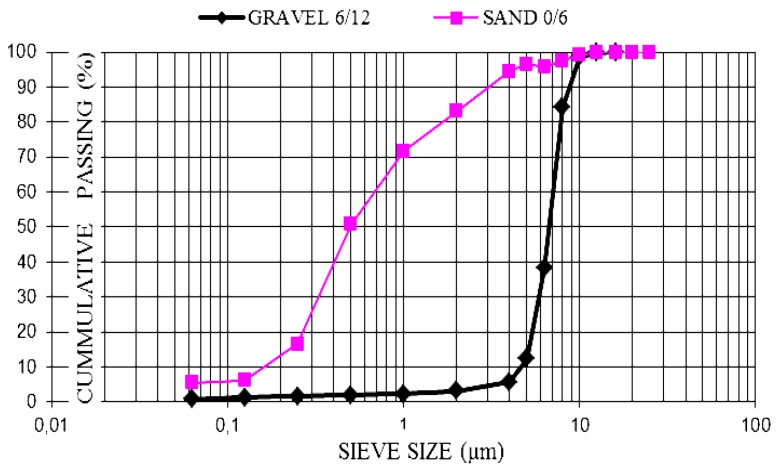
Aggregate size distribution.

Aggregates: aggregate sizes 0/6 and 6/12 were chosen for greater uniformity in movement and lower risk of flow blocking, according to UNE-EN 12620: 2003 + A1: 2009 [19]. As shown in the particle size (Figure 1) and in the certificates, it is clean sand and gravel with continuous grain size and without slabs.

- Additives: additives in accordance with UNE-EN 934-2: 2010 + A1: 2012 [20].
  ○ Superplasticizer: is a water reducer additive, a transparent brown liquid with a specific weight of 1.08 g/cm^3^ and a pH of 4.3 ± 0.5, both tests conducted at 20 °C.
  ○ Viscosity modulator: specially developed for producing concrete with enhanced viscosity, superior stability and controlled bleeding characteristics, thus increasing resistance to segregation and facilitating placement.
- Water: Meeting the requirements of the Spanish Structural Concrete Code (EHE-08) [21].
- Phase change material: is an organic PCM of microscopic polymethyl methacrylate spheres containing high purity paraffin waxes. It is an aqueous dispersion comprised of microcapsules with a wax core serving as latent heat accumulator.

If the interior temperature rises above the phase change temperature, the wax melts within the microcapsules and absorbs excess heat, and if the temperature drops, the wax solidifies and the capsules release heat. 

A PCM whose transition temperature (liquid-solid) is in the temperature range in which people feel more comfortable should be chosen, and also depending on the characteristics of each region, so that the thermal inertia of concrete matches the day-night temperature cycle.

The characteristics of the product according to the manufacturer's specifications are as follows:

42% solids content (in water); pH,7–9; viscosity, 200–600 mPa·s; density, 0.98 kg/L; melting point, 23 °C; operational range, 10–30 °C; latent heat capacity, 41 kJ/kg (emulsion); overall storage capacity, 55 kJ/kg (emulsion); applications, Indoor temperature stabilitation in comfort zone and Passive & active application. 

This PCM was selected for the following reasons:

○ Melting point addequate to temperature in the comfort zone (23 °C) expected to provide the highest termal benefit.
○ It is an aqueous dispersion, unlike those used in the previous studies [14,15,16,17], so it is expected that there is less breakage problem of microencapsulated, and easier to integrate into the mix.
○ It can be used as filler having a diameter of 2 to 20 μm, so the fine contents in the mix can be reduced, thus improving the particle distribution curve.

### 2.2. Composition of the Reference Mix

The flow behavior of SCC is governed by the paste composition, paste volume, and particle size distribution of aggregates. The factors governing the behavior of SCC are determined by the appropriate mix design method [22]. In this work the initial mix was estimated according to the method used by the Polytechnic University of Catalonia [23] developed for high-strength SCC, a methodology based on the use of locally available materials and testing techniques of common usage. 

The procedure is based on a separate optimization of the composition of the paste and the granular aggregate skeleton. The paste consists of cement, filler, water and superplasticizer, while the granular skeleton is defined by the optimum ratio between sand and gravel that provides maximum compactness of the structure. In addition, the typical ranges of the SCC components specified in guidelines of EFNARC [24] were taken into account to estimate the mix composition.

The key to obtain a concrete characterized by a high cohesion and self-compacting properties lies in the correct optimization of the composition of the cement paste in general and in the combination water/superplasticizer/viscosity-modifying agent in particular. The incorporation of the viscosity-modifying agent adversely affects the fluidity of the concrete and therefore the amount of water and/or dosage of superplasticizer [25] should be increased to attain good workability.

Table 1 shows the composition of the reference mix obtained without incorporation of the PCM, the starting basic composition on which all tests have been performed, characterized by: total amount of water, 5.02 kg; % superplasticizer/cement, 1.89; W/(C + F) Ratio, 0.30. By adding the viscosity-modifying agent, the necessary proportions were modified to obtain the required self-compacting specifications.

**Table 1:** Composition of the reference mix.

Component	Description	Weight (kg)
Cement (C)	CEM I 52.5 R	8
Filler (F)	Filler A	8.73
Gravel	Granite 6/12 mm	20.68
Sand	Granite 0/6 mm	26.53
Additive	Superplasticizer	0.151
Additive	Viscosity modulator	0.029
Water (W)	Additional water	4.92
Total	Total weight	69.04

### 2.3. Mix Design by Experiments

The test methodology used [23] has evaluated the properties of the SCC obtained according to the requirements of the Spanish Code EHE-08. 

The mixing procedure carried out in the laboratory has developed in the following steps: first, gravel is added and sand put over it to prevent it from sticking to the container; these two ingredients are mixed for 1 min. Then the filler and cement are added, and all solid components are shuffled for proper homogenization for another 1 min. From this moment, approximately 90% of the total dosage of water is slowly added and mixing continues for a further 2 min until the whole mixture becomes perfectly soaked and thus proper execution of the process of hydration is ensured. After this period of time, the two additives are added, 90% of the superplasticizer and the viscosity modulator, and mixing continues for one and a half minutes to ensure that the additives were homogeneously dispersed and activated. Finally, for the reference mix, the remaining water and superplasticizer are to be rectified as needed to obtain the desired self-compacting characteristics. 

However, for mixes in which the PCM is incorporated, it will be incorporated just before the mentioned final adjustments, to expose it to the shortest possible time to the mixing process and thus preventing the rupture of the microcapsules. Control at this point is by visual inspection.

Sampling of fresh concrete, with which standard specimens were developed, was carried out according to the UNE-EN 12350-1: 2009 [26] and curing of specimens was performed in a humid chamber.

The test program has been developed in the following steps:

The first mix that has been tested contains 5% of PCM; standard kneaded with a very long kneading time and exudation was observed. The standardization of 3 min was decided, mixing after adding the superplasticizer, and one more minute, each time water or additives needed to be added.

Based on the results, the second and the third mix added 10% and 15% of PCM respectively, and the W/(C + F) ratio was increased, as well as the percentage of superplasticizer. It was found that the concrete did not flow and the recipe of the following 20% and 25% mixes were decided to be changed. Furthermore, the specimens of the 15% mix could not be demolded because of the uncured concrete.

The quantities of each material were updated for the 20% recipe, except for the cement and the viscosity modulator additive that remained constant; in particular, filler content was reduced and the content of aggregates was increased. When doing the test, it was found that reducing the filler was not enough, so the 25% recipe was reformulated by replacing all the filler with sand and gravel. It was observed that the concrete was behaving like a conventional concrete but with a lot of air entrainment and fermentation. 22 h later, 20% specimens could not be demolded. Further, 48 h later, the 25% specimens could not be demolded. 

Table 2 shows the final dosages of each component for the respective mixes and the water/(cement + filler) ratios.

**Table 2:** Mix composition.

Mix composition	Percentage of PCM in the mix					
	0%	5%	10%	15%	20%	25%
	Dosages (kg)					
CEM I 52, 5 R	8	8	8	8	8	8
Filler	8.73	8.73	8.73	8.73	6.98	0
Granite 6/12 mm	20.68	20.68	20.68	20.68	21.38	23.61
Granite 0/2 mm	26.53	26.53	26.53	26.53	27.49	32.02
Superplasticizer	0.151	0.151	0.175	0.214	0.18	0.191
Viscosity modulator	0.029	0.029	0.029	0.029	0.029	0.029
PCM	0	0.392	0.784	1.176	1.568	1.96
Added water	4.92	4.7	4.63	4.758	4.94	3.84
Total weight	69.04	69.212	69.558	70.117	70.567	69.65
Total water	5.02	5.02	5.17	5.54	5.92	5.04
W/(C + F) Ratio	0.30	0.30	0.31	0.33	0.40	0.63
% Superplasticizer/cement	1.89	1.89	2.19	2.68	2.25	2.39

### 2.4. Testing Methods

Three testing methods have been researched in this study to determine the properties of SCC, both fresh and hardened: slump flow test, density, and mechanical compressive strength:

- Slump flow test: The appropriate behavior of SCC in the fresh state requires high fluidity with enough viscosity and cohesion among the components to ensure a continuous and uniform flow throughout the mass, not showing segregation, and without causing concrete blocking between reinforcements.

In order to evaluate self-compactability, slump flow test was performed according to the UNE-EN 12350-8: 2011 [27], by determining flow (d_f_) and flow time (T_50_), measured by leaving concrete flowing freely after placing it into a cone-shaped mold. We performed a slump flow test for each mix.

- Density: In order to determine density, a total of six samples were analyzed for each dosage, according to UNE-EN 12350-6: 2009 [28] (fresh concrete) and UNE-EN 12390-7: 2009 (hardened concrete) [29].
- Compressive strength: It was tested by 15 cm × 30 cm cylindrical specimens of each dosage calculating the 28 days strength according to the UNE-EN 12390-3: 2009/AC: 2011 [30]. To make a more comprehensive assessment, strength was calculated at seven and 60 days.
- Thermal conductivity: It was determined by means of tests according to the ASTM C 114-06 [31] standard with Thasys specimens, sheet materials with a size of 70 by 100 mm and of 7 mm ± 1 mm thickness.

We conducted a first test to the reference sample, containing no PCM, and once, selected the sample with the highest percentage of PCM that meets the required specifications, tests are performed to measure the thermal conductivity.

## 3. Results and Discussion

### 3.1. Slump Flow Test

Table 3 shows the results of the tests performed, as well as their classification according to EFNARC. The first thing to be noticed is that the test could not be performed for the 25% mix, so the spread circle diameter was measured in the Abrams cone resulting in a d_f_ = 220 mm, which is not within the acceptable range. The mix of 20% does not fulfill slump flow, as the permissible range for that parameter is 550 mm < d_f_ < 850 mm. As a result of the slump flow analysis, all measures are in a narrow range but are classified in different groups, SF2 and SF1, because the range cut is 650 mm.

In general, all mixes have good properties and it can be seen that the increasing percentage of PCM in the mix decreases the deformability of SCC (d_f_ decreases), and similarly (except for the 10% mix) with viscosity and cohesion (T_50_ decreases). The T_50_ parameter alone cannot represent viscosity and can only score it when the value of the flow extension is constant in the evaluated mixes [25]. In our case, it is useful for the relative evaluation of the 10% and 15% mix viscosity, possessing identical d_f_. The mix of 10% presents a longer time because of its composition; which implies increased viscosity and cohesion than the 15% mix. This is because the contribution of water is lower for similar PCM and superplasticizer increases.

**Table 3:** Slump flow test results and self-compacting concrete (SCC) classification according to Annex A of the EFNARC.

Test	Parameter	Percentage of PCM in the Mix					
		0%	5%	10%	15%	20%	25%
Slump flow test	T_50_ (s)	8	7	8	6	8	–
	d_f_ (mm)	690	650	640	640	530–520	Cone 22 cm
Classification according to EFNARC	Viscosity	VS2	VS2	VS2	VS2	VS2	–
	Slump flow	SF2	SF1	SF1	SF1	–	–

It should be mentioned that the results of this study do not provide a direct measure of the assessment of the ready-mixed concrete to pass easily between the reinforcement of the structural panel to be made. However, since there is slight reinforcement in this type of product, no risk of blockage is deemed, as such no further test has been considered necessary. 

On the other hand, slump flow test does allow to assess qualitatively in visual terms, the presence of segregation appreciable in the 5% mix; which is why the amount of added water was decided to be reduced, and the percentage of superplasticizer increased. In the remaining samples, we observed a uniform distribution of coarse aggregate and no segregation or exudation on the perimeter of the final spread circle of the test.

### 3.2. Density

Table 4 shows the values obtained for the density of each sample, m_1_ being the container mass in kilograms, m_2_ the container mass plus the mass of the mortar in the container in kilograms, m_24h_ is the difference between m_2_ after 24 h and m_1_ in kilograms, D is the fresh concrete density in kilograms per cubic meter, and D_24h_ is the density after 24 h in kilograms per cubic meter for the same volume.

**Table 4:** SCC densities according to the percentages of added phase change material (PCM).

Mix	m_1_ (kg)	m_2_ (kg)	m_2_ − m_1_(kg)	m_24h_ (kg)	D (kg/m^3^)	D_24h_ (kg/m^3^)
0% PCM	1.190	9.230	8.040	7.995	2382	2369
	1.145	9.235	8.090	8.030	2397	2379
	1.200	9.250	8.050	7.995	2385	2369
	1.150	9.245	8.095	8.035	2399	2381
	1.185	9.265	8.080	8.010	2394	2373
	1.185	9.250	8.065	8.005	2390	2372
5% PCM	1.235	9.265	8.030	7.965	2379	2360
	1.220	9.285	8.065	7.990	2390	2367
	1.165	9.225	8.060	7.995	2388	2369
	1.205	9.270	8.065	8.000	2390	2370
	1.190	9.190	8.000	7.945	2370	2354
	1.190	9.260	8.070	8.010	2391	2373
10% PCM	1.185	9.050	7.865	7.810	2330	2314
	1.140	9.045	7.905	7.855	2342	2327
	1.195	9.040	7.845	7.805	2324	2313
	1.145	9.020	7.875	7.830	2333	2320
	1.175	9.075	7.900	7.835	2341	2321
	1.175	9.020	7.845	7.875	2324	2333
15% PCM	1.225	8.905	7.680	7.580	2276	2246
	1.120	8.910	7.790	7.630	2308	2261
	1.160	8.930	7.770	7.700	2302	2281
	1.200	8.975	7.775	7.705	2304	2283
	1.190	8.965	7.775	7.700	2304	2281
	1.190	9.000	7.810	7.730	2314	2290
20% PCM	1.145	8.915	7.770	7.675	2302	2274
	1.185	8.890	7.705	7.695	2283	2280
	1.200	8.920	7.720	7.665	2287	2271
	1.145	8.905	7.760	7.710	2299	2284
	1.180	8.935	7.755	7.705	2298	2283
	1.180	8.940	7.760	7.705	2299	2283
25% PCM	1.225	7.950	6.725	6.760	1993	2003
	1.105	7.935	6.830	6.680	2024	1979
	1.170	8.055	6.885	6.805	2040	2016
	1.170	8.070	6.900	6.795	2044	2013
	1.230	7.995	6.765	6.745	2004	1999
	1.115	7.975	6.860	6.640	2033	1967

**Figure 2:**
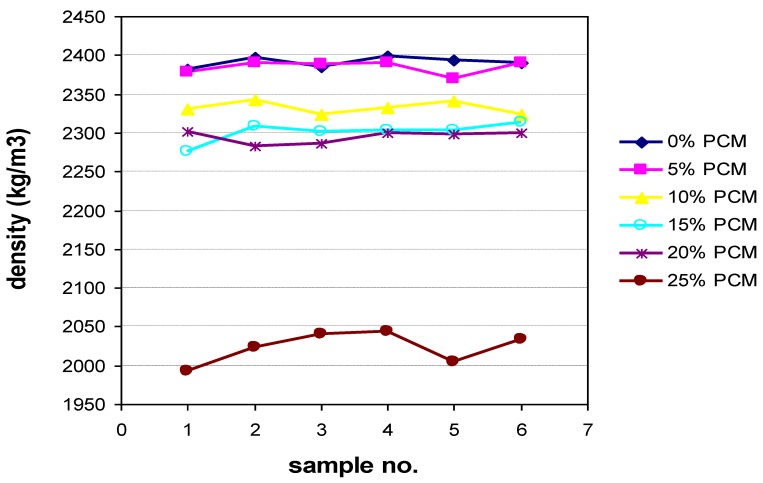
Densities by percentage of added PCM.

Figure 2 shows the density ratio of each sample to the percentage of added PCM, where you can see the consistency of the data and the large decrease in density for the sample with 25% of PCM. Problems already occurred with that sample in the slump flow test. 

**Figure 3:**
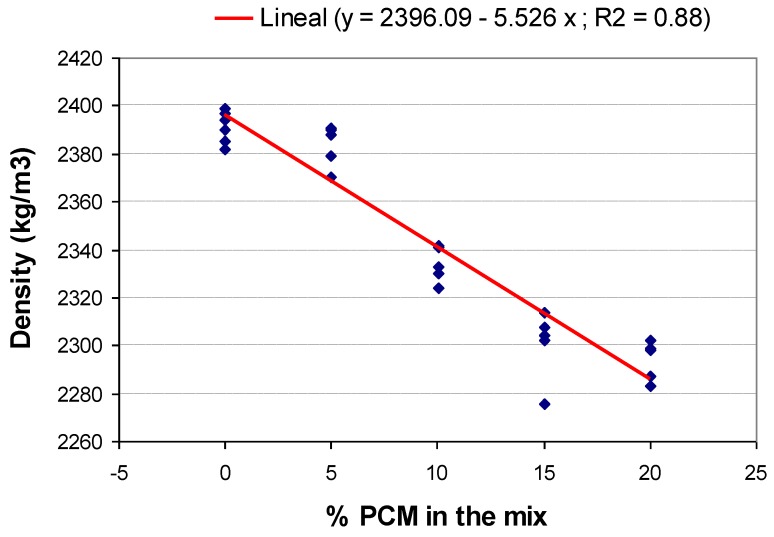
Density ratio to percentage of PCM.

The scatter diagram (Figure 3), wherein the 25% sample was discarded for the reasons mentioned above, proofs the inverse linear relation between the density and percentage of PCM variables present in the sample, providing a reduction of 1.1% in the density for each 5% of added PCM. This linear relation holds for the range of PCM contents considered.

In order to further clarify the results obtained, Table 5 shows the average values of those densities in fresh and after 24 h, as well as the difference between them. 

**Table 5:** Average densities.

Mix	ρ_fresh_ (kg/m^3^)	ρ_24h_ (kg/m^3^)	ρ_fresh_ − ρ_24h_ (kg/m^3^)
0% PCM	2391	2374	17
5% PCM	2385	2366	19
10% PCM	2333	2321	11
15% PCM	2301	2274	27
20% PCM	2295	2279	16
25% PCM	2023	1996	27

The results shown reflect how both fresh and 24 h density is proportionately reduced as the percentage of PCM is increased in the sample. This decrease is more than evident in the 25% mix, at which the density is reduced by 13.4% compared to the previous mix, mainly due to the total replacement of the filler by PCM, which causes a high W/(C + F) ratio. 

What is also evident from Table 5 is that the incorporation of the PCM implies a worse homogeneity of the mix, resulting in pores with occluded air. The 10% mix shows the best curing behavior, losing only 11 kg/m^3^ by evaporation through these pores. The 15% and 25% of PCM mixes show the worst behaved, losing up to 27 kg/m^3^ of water.

Initial specifications of structural panel to be manufactured indicate an approximate density of 2400 kg/m^3^. In view of the results, the densities obtained are slightly lower, with values in fresh ranging between 2023 and 2391 kg/m^3^. Thus, it was decided not to accept densities below 2300 kg/m^3^ which imply that the level of blocked air is very high, with the appearance of holes and cavities that causes a significant decrease in compressive strength and a high risk that the durability of concrete may be affected. Therefore, mixes with 15%, 20%, and 25% are ruled out due to lower densities in fresh.

### 3.3. Compressive Strength

**Figure 4:**
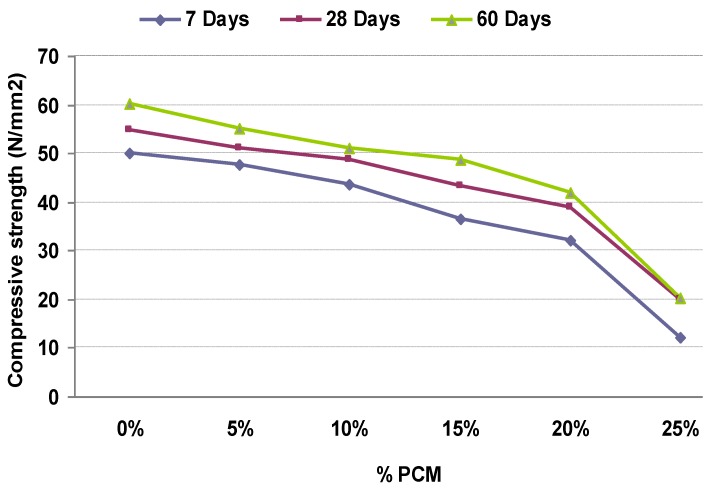
Relation between mechanical compressive strength and percentage of added PCM.

**Figure 5:**
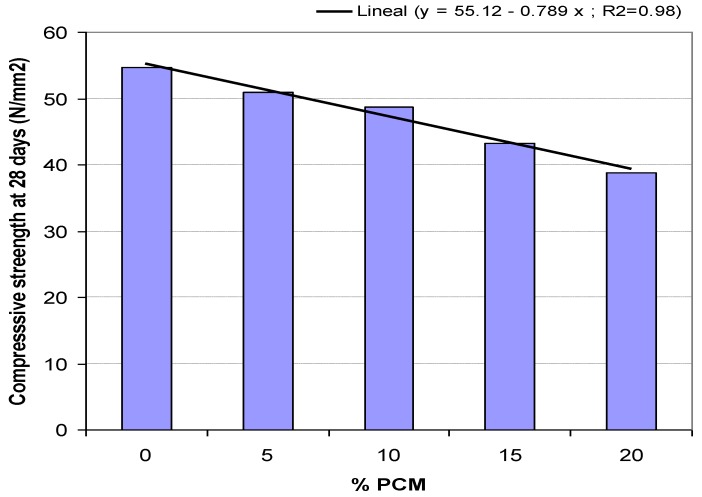
Mechanical compressive strength at 28 days.

Table 6 shows the values of the test. Figure 4 represents a comparative analysis of compressive strength in terms of the percentage of added PCM, and Figure 5 represents the linear relation between the compressive strength at 28 days and the percentage of PCM, rejecting the sample with 25% for the problems detected that makes it not representative for this study.

**Table 6:** Mechanical compressive strength.

**Specimen Breakage**	**Percentage of PCM**					
	0%	5%	10%	15%	20%	25%
	**Compressive strength (N/mm2)**					
7 days	50.02	47.70	43.65	36.40	32.20	12.10
28 days	54.70	51.00	48.65	43.25	38.85	20.10
60 days	60.10	55.00	51.20	48.60	42.05	20.20

Strength decreases as the percentage of PCM added to the sample increases; this is corroborated by the results obtained in previous studies [14,15,17]. Compressive strength, as highlighted in the analysis of densities, greatly wanes in the mix with 25% of PCM, reducing 20 N/mm^2^. Figure 5 also concludes that the compression strength decreases by 7% for each 5% added PCM. This linear relation is maintained for the considered parameter range. 

**Figure 6:**
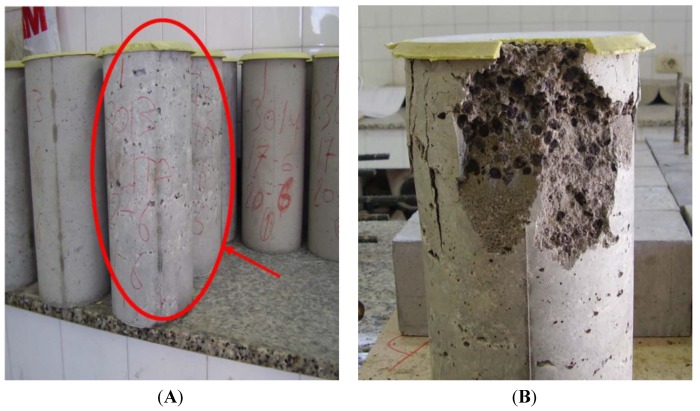
(**A**) Specimens of mixes; (**B**) Breakage of a specimen with 20% PCM.

Figure 6A shows the specimens with bonded sulfur mortar caps waiting for the test, and the cavities problems mentioned above for the blends of 20% and 25% are visible at a glance. Such problems become more evident after the test (Figure 6B), where we can see an irregular breakage of the specimen and poor homogenization of the mixture, with many pores highlighting the lack of compaction. 

An additional problem shown in this specimen is segregation of the PCM particles to the upper surface of the concrete during curing, because its density is lower than that of ceramics and are also of very small size, so that the particles diffuse upwardly through the liquid, since the viscosity of this sample is unable to halt it; the aggregation of particles that huddle forming clusters can be clearly seen. This occurs when dispersing solids and liquids of different polarity, as is the current case (the liquid is water, polar, and the PCM is polymeric, non-polar).

**Figure 7:**
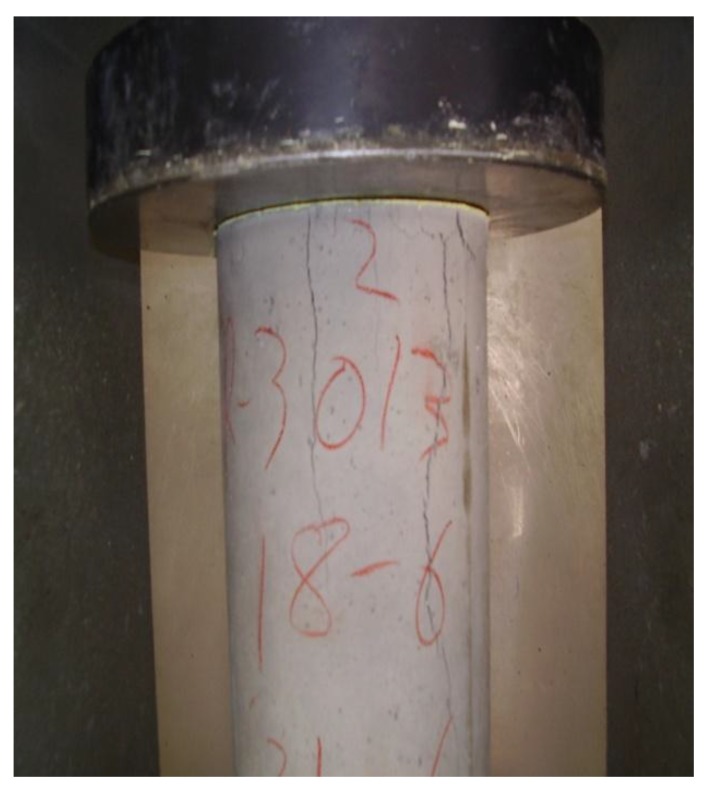
State of cracking of the sample with 10% of PCM during the trial.

Figure 7 relates to a compression test of a specimen with 10% of PCM. It is clearly seen that the surface is uniform with good surface finish and good performance of cracking. 

Due to the results discussed above, and considering that the structural panel to be manufactured must have a characteristic strength at 28 days of 40 N/mm^2^, the viability of samples with 20% and 25% percentages of PCM are discarded because of lower strength.

### 3.4. Thermal Conductivity

The test conditions and the characteristics of the specimens are shown in Table 7.

**Table 7:** Characterization of specimens and test conditions.

Property/Condition	0% PCM Mix	10% PCM Mix
Average thickness of specimens (mm)	7.45	6.98
Average weight of specimens (g)	129.40	119.29
Thermal conductivity (W/m·K)	1.79 ± 0.05	1.35 ± 0.04

A decrease in thermal conductivity can be clearly observed when PCM is added; such decrease is expected because of the reduction in the weight of the specimens, backed up by the results obtained in the determination of densities. This reduction is brought about by the amount of air entrainment and the low thermal conductivity of the paraffin.

The result, a reduction by 25%, has been better than expected regarding the initial objectives (thermal conductivity of 1.5 W/m·K), and in terms of experimental results obtained in studies of [15] and [17]. This shows that thermal conductivity depends not only on the percentage of PCM, but also on the average size of the particles in the composition of the mix. The fact that an aqueous dispersion of PCM was used in this investigation makes it easier to integrate into the mixture and that the design of the mix is optimal.

## 4. Conclusions 

The main objective of this research is to develop a structural panel, which allows energy savings, with the economic and environmental improvements that it entails, besides contributing to the comfort of the users of the buildings and facilities where they are used.

The precast concrete panel, a HA-40/AC/12/IIa, should have an approximate density of 2400 kg/m^3^. The work presented here is based on the design of SCC mixes with different additions of microencapsulated PCM (0%, 5%, 10%, 15%, 20%, and 25% of Micronal DS 5007 X in aqueous dispersion).

Based on tests and subsequent analysis of the different mixes, we can conclude that as the percentage of PCM in the mixture increases:

1. Deformation capacity decreases as well as the concrete viscosity and cohesion.
2. Density in fresh decreases by 1.1% per every 5% of added PCM.
3. Compressive strength decreases by 7% for every 5% of added PCM.
4. Thermal conductivity decreases by 25% in the mix with 10% of PCM.

In addition to these general considerations, self-compactability problems have been found in samples with 20% and 25% of PCM, as well as segregation in the sample with 5% of PCM; problems of poor finishes and hollows with poor sample homogenization in the specimens containing 20% and 25% of PCM, and segregation of PCM particles to the upper surface. Due to practical considerations, the limit of density has been established at 2300 kg/m^3^ so the mix with 10% offers the highest permitted percentage. Definitively, the best results for the demanded technical specifications of the panel to be manufactured have been observed in the mix with 10%, so we can conclude that this is the maximum permitted percentage.
